# Embryonic vascular endothelial cells are malleable to reprogramming via Prox1 to a lymphatic gene signature

**DOI:** 10.1186/1471-213X-10-72

**Published:** 2010-06-28

**Authors:** Harold Kim, Vicky PKH Nguyen, Tatiana V Petrova, Maribelle Cruz, Kari Alitalo, Daniel J Dumont

**Affiliations:** 1 Sunnybrook Research Institute University of Toronto 2075 Bayview Avenue Toronto, Ontario, M4N 3M5, Canada; 2 Biomedicum Helsinki Haartman Institute PO Box 63 (Haartmaninkatu 8) FI-00014 University of Helsinki, Helsinki, Finland; 3 Division of Experimental Oncology CePO, CHUV and University of Lausanne 155, Chemin des Boveresses CH-1066 Epalinges Switzerland

## Abstract

**Background:**

*In vivo *studies demonstrate that the Prox1 transcription factor plays a critical role in the development of the early lymphatic system. Upon Prox1 expression, early lymphatic endothelial cells differentiate from the cardinal vein and begin to express lymphatic markers such as VEGFR-3, LYVE-1 and Podoplanin. Subsequent *in vitro *studies have found that differentiated vascular endothelial cells can be reprogrammed by Prox1 to express a lymphatic gene profile, suggesting that Prox1 can initiate the expression of a unique gene signature during lymphangiogenesis. While the *in vitro *data suggest that gene reprogramming occurs upon Prox1 expression, it is not clear if this is a direct result of Prox1 in vascular endothelial cells *in vivo*.

**Results:**

Overexpression of Prox1 in vascular endothelial cells during embryonic development results in the reprogramming of genes to that of a more lymphatic signature. Consequent to this overexpression, embryos suffer from gross edema that results in embryonic lethality at E13.5. Furthermore, hemorrhaging and anemia is apparent along with clear defects in lymph sac development. Alterations in junctional proteins resulting in an increase in vascular permeability upon Prox1 overexpression may contribute to the complications found during embryonic development.

**Conclusion:**

We present a novel mouse model that addresses the importance of Prox1 in early embryonic lymphangiogenesis. It is clear that there needs to be a measured pattern of expression of Prox1 during embryonic development. Furthermore, Prox1 reprograms vascular endothelial cells *in vivo *by creating a molecular signature to that of a lymphatic endothelial cell.

## Background

The specialization of the vasculature is driven by a number of molecular pathways that dictate the fate of blood vessels to that of an artery or vein [1]. Following this initial programming, the development of the lymphatic system is dependent on the further differentiation of the venous endothelium. The newly differentiated lymphatic endothelial cells migrate in a polarized fashion to coalesce into an early lymph sac, which then progress to form the lymphatic vasculature proper [2,3].

A number of genes have been found to be associated with the lymphatic endothelial cell profile, for example LYVE-1, Podoplanin and VEGFR-3 [4-6]. In addition to this profile, the transcription factor Prox1 has been found to act as an important regulatory switch, altering the molecular identity of venous endothelium and imparting them with attributes that augment lymphatic development. Indeed, genetic ablation of *prox1 *results in embryonic lethality; hemizygous null mice are found to die shortly after birth [3]. In both cases, a major developmental defect was found to involve the emerging lymphatic system. Specifically, the early lymphatic endothelial cells (LECs) that normally bud off from the cardinal vein (CV) in a polarized manner do not do so. Instead, *prox1 *null LECs do not migrate, resulting in defective lymph sac formation and consequently no development of a functional lymphatic system. Furthermore, these null cells molecularly default to a more vascular-like phenotype. To this end, *prox1 *null mice display lymphedema and chylous ascites buildup resulting in death [2,3].

It is clear that vascular endothelial cells require the regulated expression of Prox1 early in development, initiating a transcriptional program that results in lymphatic differentiation. This point underscores the plasticity of differentiated endothelial cells under the influence of Prox1. Indeed, microarray analysis of vascular endothelial cells engineered to overexpress Prox1 resulted in the upregulation of lymphatic specific genes while downregulating a number of blood specific genes [7,8]; this study extending the known surface markers of lymphatic endothelial cells by providing a more in-depth characterization of LEC gene signature. The *in vivo *knockout data demonstrates that *prox1 *plays an important role in early lymphatic development. Furthermore, targeted deletion of *prox1 *within vascular endothelial cells recapitulates the lymphatic defect and the edematous phenotype [9]. Moreover, *in vitro *studies suggest that this lymphatic defect is potentially due to an inability to molecularly reprogram early vascular endothelial cells to a lymphatic fate [7,8]. Given this however, it is not clear whether the reprogramming to a specific gene signature does indeed occur *in vivo*. Here we describe an *in vivo *model to address the molecular significance of Prox1 in reprogramming vascular endothelial cells to that of a lymphatic gene profile.

## Results

### Prox1 expression in double transgenic mice

**Figure 1:**
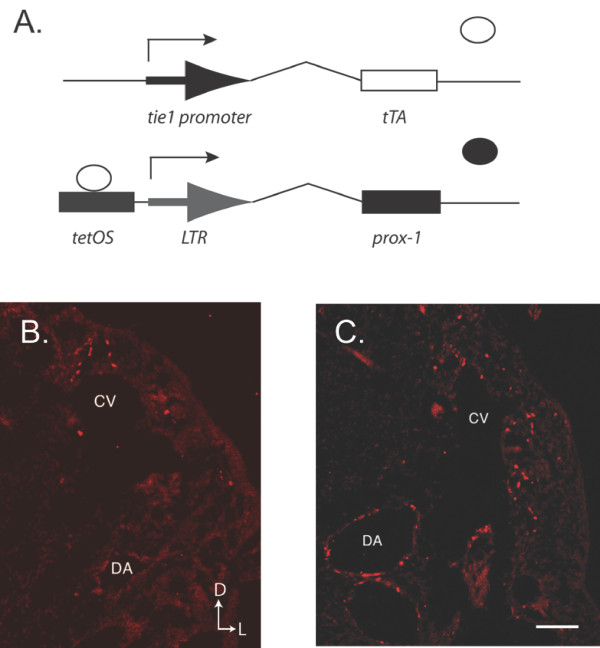
**Bigenic model for Prox1 overexpression**. (A) The *prox1:tetOS *transgene is driven by the tissue specific *tie1:tTA *promoter. Open circle: tTA, closed circle: Prox1 protein. (B) The expression of Prox1 in E10.5 embryos displays a polarized expression pattern specifically from the cardinal vein. (C) In contrast, overexpression of Prox1 in the double transgenic (DT) embryos display an increase in Prox1 positive cells from the cardinal vein. Significantly, Prox1 is also present on the dorsal aorta, which is normally negative for Prox1 expression. Orientation arrows for B and C: D- Dorsal, L- Lateral. Scale bar: 50 μm.

In order to investigate whether Prox1 is necessary and sufficient in regulating cell fate, we have generated a mouse model that forces the expression of Prox1 specifically in vascular endothelial cells. Our transgenic model takes advantage of a bigenic expression system driven by the *tie1 *promoter (Figure 1a). This model results in targeted expression within vascular compartments such as the cardinal vein as well as the dorsal aorta (Figure 1b and 1c, see Additional file 1). While the expression of Prox1 in DT embryos is also found on both arteries and veins, both these vascular structures appear to differentially overexpress Prox-1 throughout development in our model suggesting that arterial and venous endothelial cells may regulate Prox-1 RNA/protein levels in a different fashion. While the directionality of the budding lymphatic endothelial cells appear to be more tightly regulated in control embryos, DT embryos appear to display more Prox1 positive cells in the periphery. These results suggest that normally, Prox1 expression and lymphatic endothelial cell budding are tightly controlled and can be disrupted by the overexpression of Prox1 in the developing vasculature.

### Phenotype of DT Prox1 mice

**Figure 2:**
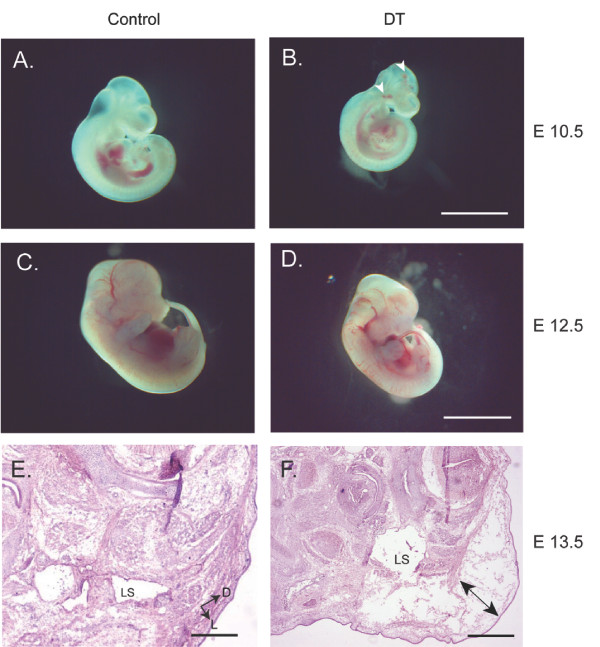
**Overexpression of Prox1 results in embryonic lethality at E13.5**. Control and DT embryos were analyzed at (A and B) E10.5 and (C and D) E12.5. Interestingly, overexpression of Prox1 results in a small amount of hemorrhaging and growth retardation (B; arrowheads). Later time points display exacerbated bleeding with a consistent delay in growth (C and D). (E and F) Histological analysis at E13.5, embryos display gross edema typified by separation of the dermis from the epidermis (F; double arrow). Also significant is the increase in lymph sac size (LS) in Prox1 transgenic embryos. LS: Lymph sac. Orientation arrows for E and F: D- Dorsal, L- Lateral. Scale bars for A and B: 5.0 mm; C and D: 1.0 cm; E: 250 μm and F: 500 μm.

As development progresses, both the vascular and lymphatic system matures to form two separate yet critically interrelated circuits. In early development, there are no overt deficiencies in the blood vasculature of DT embryos. However, by E10.5 we begin to observe small hemorrhages on the surface of developing DT embryos (Figure 2a and 2b, arrorwheads). At later stages in development we notice some subtle delays in growth, more profound blood loss, with blood pooling within the embryo (Figure 2c and 2d). In the most severe embryonic cases we find anemic embryos that display massive edema at E13.5 where the epidermis separates from the body as a result of trapped extravasated fluid (Figure 2e and 2f, double arrowhead). Upon further analysis, DT embryos that harbor the most severe phenotypes also display enlarged lymph sacs relative to their control counterparts (Figure 2e and 2f). These results suggest that the overexpression of Prox1 can pose a developmental crisis during early gestation. Indeed, 33% of postnatal DT's do not make the expected Mendelian double transgenic ratio (Table 1). However, the majority of the postnatal DT's do appear to survive to birth. These results suggest that the overexpression of Prox1 can have deleterious consequences in the developing embryo correlating with hemorrhaging, anemia and edema.

**Table 1:** Analysis of number of embryos and postnatal births.

*E10.5*				
+/+ 70	Prox1/+ 58	Tie1/+ 65	Tie1/Prox1 46	Total 239
DT observed*19.2% DT expected 25% Ratio of observed:absent**(%) 76:24				
*E12.5*				
+/+ 83	Prox1/+ 34	Tie1/+ 52	Tie1/Prox1 29	Total 198
DT observed 14.6% DT expected 25% *Ratio of observed:absent (%) 58:42*				
*E13.5*				
+/+ 35	Prox1/+ 9	Tie1/+ 29	Tie1/Prox1 9	Total 82
DT observed 10.9% DT expected 25% *Ratio of observed:absent (%) 44:56*				
*Postnatal*				
+/+ 68	Prox1/+ 54	Tie1/+ 57	Tie1/Prox1 36	Total 215
DT observed 16.7% DT expected 25% *Ratio of observed:absent (%) 67:33*				

* *DT observed % is calculated as the number of DT animals/Total number of animals × 100%*.

** *Observed % is calculated as the DT observed %/DT expected% = percentage of animals observed for that Mendelian ratio × 100%. Absent % is calculated as 100-observed%*

A catalogue of the total number of embryos and live births from each genotype at various developmental timepoints. Also shown is the percentage of DT births observed and expected, with the % ratio of observed births versus the percentage presumed to be lethal. Total of 519 embryos were assessed and 215 postnatal births observed.

### Marker analysis of yolk sacs from Prox1 DT mice

**Figure 3:**
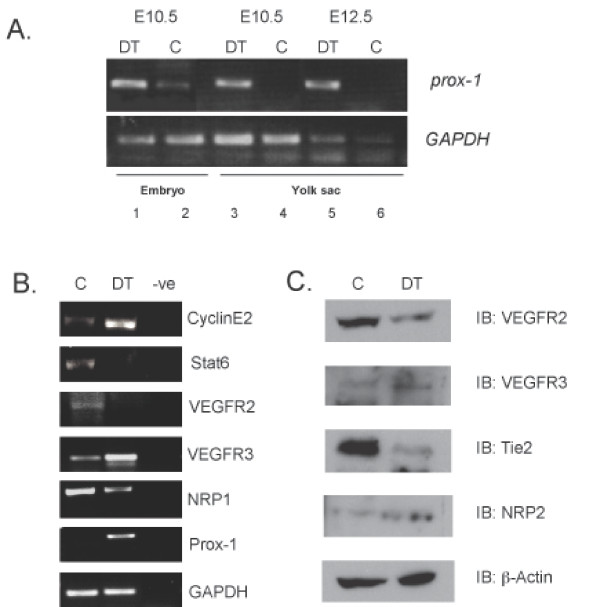
**Analysis of reprogramming in yolk sacs from Prox1 double transgenic embryos**. (A) Analysis of control and DT embryos at E10.5 (lanes 1 and 2) and yolk sacs at E10.5 or E12.5 (lanes 3 to 6) for *prox1 *expression by RT-PCR. (B) Yolk sacs from E13.5 control and DT embryos were analyzed using RT-PCR for the expression of lymphatic associated markers such as CyclinE2, Stat6, VEGFR-2, VEGFR-3 and Neuropilin-1 (NRP1). -ve represents no template control. (C) Similarly, yolk sacs from E12.5 control and DT embryos were analyzed by western blot for the expression of VEGFR-2, VEGFR-3, Tie2 and Neuropilin-2 (NRP2).

Given the influence of Prox1 on gene regulation in vascular and lymphatic endothelial cells, we further investigated the possibility of a switch in the molecular identity of endothelial cells using markers that are influenced by Prox1. To this end, we started our analysis with the yolk sac, a relatively simple tissue that is rich in blood vasculature (Figure 3a). Analysis of control or DT embryos reveal that the overexpression of Prox1 in vascular endothelial cells could alter gene expression to that of a more lymphatic endothelial cell signature. For example, transcripts for genes such as VEGFR-3 and CyclinE2 increase while VEGFR-2, Neuropilin1 and Stat6 decrease (Figure 3b). Furthermore, protein levels from yolk sacs derived from DT embryos demonstrate that VEGFR-2 and Tie2 decrease while Neuropilin-2 increases with a marginal increase in VEGFR-3. Wholemount analysis of DT yolk sacs support the reprogramming event, indicated with an increase in Podoplanin expression that correlates with Prox1 overexpression (data not shown). Consistent with previous findings, the overexpression of Prox1 in vascular endothelial cells appear to sufficiently alter the gene signature of BECs to that of a more LEC profile *in vivo*.

### Jugular veins from Prox1 DT embryos display lymphatic markers

**Figure 4:**
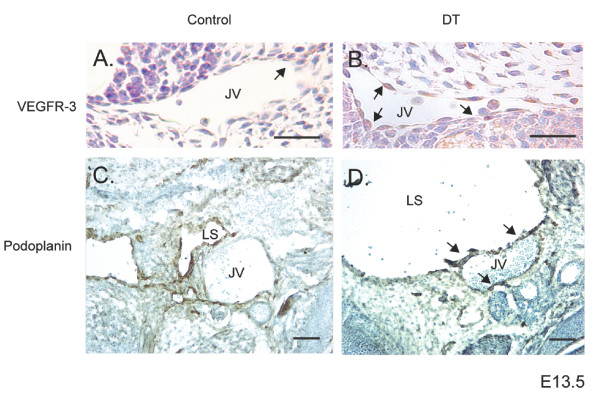
**Immunohistochemistry of Prox1 embryos for lymphatic markers**. E13.5 embryos were analyzed for the markers VEGFR-3 and Podoplanin on the jugular vein (JV) of control and double transgenic Prox1 embryos. (A) In control samples VEGFR-3 expression is relatively low in comparison to (B) Prox1 DT embryos (arrows). (C) In control embryos, Podoplanin is found expressed on the jugular vein during early development, however it begins to be downregulated and moves to the lymph sac (LS). (D) In contrast, overexpression of Prox1 results in the sustained expression of Podoplanin on the jugular vein (JV; arrowheads) as well as the enlarged lymph sac (LS). Scale bar: 100 μm.

To visualize the potential of Prox1 in reprogramming the vascular endothelium, markers such as VEGFR-3 and Podoplanin were used to analyze the developing jugular vein (JV). Within the DT samples, it was found that the levels of VEGFR-3 increased relative to that observed in the control embryos (Figure 4a versus b, arrows). Previous studies by Schacht et al. show that Podoplanin expression is tightly regulated during development, being expressed early on the JV and gradually being downregulated by E13.5. This downregulation coincides with a shift in expression to the developing lymph sacs derived from the same JV [5]. Interestingly, at the timepoint where Podoplanin expression should be downregulated on the jugular vein, DT embryos show strong expression of Podoplanin relative to controls (Figure 4c versus d, arrows). These results further suggest that the overexpression of Prox1 can alter the expression pattern of vascular endothelial cells to that of a more lymphatic signature.

### Overexpression of Prox1 correlates with changes in junctional proteins in vivo and in vitro

**Figure 5:**
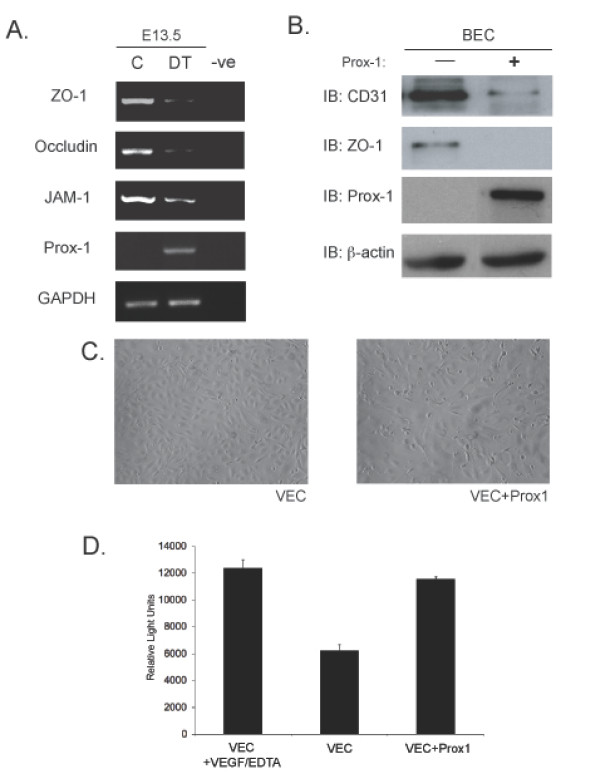
**Overexpression of Prox1 results in changes in junctional proteins in vivo and in vitro**. (A) RT-PCR analysis was performed on yolk sacs derived from E13.5 embryos to targets such as ZO-1, Occludin and JAM-1. (B) Similarly, *in vitro *analysis shows that overexpresion of Prox1 in vascular endothelial cells decreases junctional protein levels. (C) Bright field microscopy of VECs transfected with Prox1 (10x mag) (D) Vascular endothelial cells that overexpress Prox1 display an increase in permeability measured by FITC-albumin transit in a Boyden chamber assay. Figure is representative of three experiments with all samples run in triplicate. Error bars represent standard deviation of the mean. *P *< 0.05.

With the overexpression of Prox1, changes in gene signatures are observed in the developing embryo. These changes correlate with defects in embryonic development such as edema and hemorrhaging. While it suggests that the overexpression of Prox1 in the early developing vasculature is causal to these defects, the functional explanation for the edema and hemorrhaging is unclear. One possibility that may explain the observed developmental defects may lie in changes in vessel permeability upon Prox1 overexpression. To investigate this possibility, proteins that are associated with tight junctions were analyzed from yolk sacs of control and DT embryos. Interestingly, the overexpression of Prox1 results in the alteration of junctional proteins from the normal pattern of expression such as ZO-1, Occludin and to a lesser extent JAM-1 (Figure 5a). Further analysis using cultured venous endothelial cells [10] show that overexpression of Prox1 results in a similar trend of misregulated junctional targets such as ZO-1 and PECAM-1 (Figure 5b), these changes correlating with an altered cellular morphology (Figure 5c). To further define the consequences of Prox1 overexpression in VECs, an *in vitro *permeability assay via Boyden chamber was performed comparing control VECs with those that overexpress Prox1. Significantly, VECs that overexpress Prox1 display a higher degree of permeability, assessed by the passage of FITC-albumin, when compared to control VECs (Figure 5d). This suggests that VECs become more permeable with Prox1 overexpression, potentially due to alterations in the integrity of cell-cell junctions.

## Discussion

The importance of Prox1 in initializing differentiation was shown to be a result of its ability to reprogram blood endothelial cells to a lymphatic endothelial cell profile. This molecular switch shown *in vitro *suggested that Prox1 initiates the transcriptional machinery necessary for lymphangiogenesis [8,11]. While the Prox1 knockout study as well as a tissue specific deletion of Prox1 in the endothelium [9] point to the necessity of Prox1 in lymphatic development, it does not provide a complete extension on the molecular players that confer the ability to reprogram the blood vasculature as suggested from *in vitro *studies [7,8]. To this end, one way to address this involves the specific overexpression of Prox1 in the endothelial cell compartment. In the present study, we examine the importance of vascular reprogramming and fate specificity *in vivo *with a *tie1/prox1 *bigenic mouse model that specifically expresses Prox1 in early vascular endothelial cells.

Early expression of Prox1 in vascular endothelial cells result in no overt embryonic phenotype, however as development progresses Prox1 double transgenic embryos appear growth delayed and display more overt bleeding. Of the most serious cases, DT embryos are anemic and edematous. This correlated with the alteration of a number of molecular targets associated with vascular reprogramming such as an increase in VEGFR-3, Neuropilin-2 and STAT6 and a decrease in Neuropilin-1, VEGFR-2, Tie-2 and CyclinE2 [7,8,12]. Examination of the most severely affected DT embryos show a general degradation of the embryonic structure, separation of the epidermis and enlarged lymph sacs when compared to their wild type counterparts. Furthermore, vascular structures such as the jugular vein, which normally express low levels of VEGFR-3 and Podoplanin are now highly positive for these lymphatic markers. In conjunction with previous data demonstrating the importance of Prox1 in BEC differentiation to LECs, we present data that provides further *in vivo *evidence of the molecular players involved in the reprogramming of BECs to LECs early in lymphatic vascular development. Consistent with our data, it was recently observed that Sox18 is upstream of Prox1; *in vitro *overexpression of Sox18 in embryonic stem cells and vascular endothelial cells results in the upregulation of Prox1 and an increase in lymphatic markers such as Podoplanin, indirectly confirming our data presented here [13]. In addition, the conditional deletion of Prox1 results in the dedifferentiation of lymphatic endothelial cells to that of a more vascular endothelial cell-like phenotype [14]. This further suggests that Prox1 is critical for the maintenance of the lymphatic endothelial cell fate, and that endothelial cells are genetically malleable. Our *in vivo *study further confirms the importance of Prox1 in generating a lymphatic profile by altering the gene signature of vascular endothelial cells.

One consequence that results from the overexpression of Prox1 in the vascular compartment of the developing embryo is edema. At the histological level, we observe the separation of the dermal layer from the epidermis as a result of fluid leakage, characteristic of an improper lymphatic drainage system. Furthermore, a prominent feature of the most severely affected embryos is the enlargement of the lymph sacs. It is unclear as to the mechanism of the increased size, whether it is from an increase in the number of differentiating early lymphatic endothelial cells budding from the jugular vein, or from an increase in proliferation of committed lymphatic endothelial cells that are in the process of or have already formed the lymph sac. Interestingly, early analysis of E10.5 DT embryos show an increase in Prox1 positive cells in the periphery; this increase in population may play a contributing factor to the increase in lymph sac size. The potential for an increase in proliferation due to Prox1 overexpression is consistent with previously published work that identifies the upregulation of a number of cell cycle targets such as CyclinE2 and p57*kip *[7].

The data suggests that the overexpression of Prox1 during early embryogenesis results in the reprogramming of vascular endothelial cells to a more lymphatic cell fate resulting in edema, hemorrhaging and death. While it demonstrates the importance of tightly regulating Prox1 in development, it is unclear as to why the hemorrhaging occurs. In our model, one can hypothesize that the overexpression of Prox1 may influence junctional proteins involved in maintaining the integrity of cell-cell contacts; alterations in these same complexes may result in changes in vascular permeability leading to a more permeable, lymphatic-like state [15]. Indeed, upon Prox1 overepression changes in junctional proteins are observed away from the normal molecular profile associated with control embryos. Previous studies have shown that increases as well as decreases in junction protein levels have been shown to be associated with an increase in permeability [16,17], suggesting that a balance is required in order to maintain the integrity of the cell-cell interface. Recent data has found that the overexpression of Prox1 in colon epithelial cells result in dysplasia and transformation that correlates with changes in cellular adhesion [11]. Furthermore, conditional deletion of Prox1 results in aberrant junctional complex formation and abnormal pericyte association [14]. These lines of evidence point to the importance of cell-cell integrity in the regulation of vascular permeability. Moreover, the controlled expression of Prox1 is critical for normal lymphatic development in the embryo.

It is clear that the measured expression of Prox1 as well as other lymphangiogenic factors during embryonic development is essential for the proper formation of the lymphatic vasculature. Not surprisingly, compromising this regulated expression has been found to result in negative clinical consequences. For example, the growth of lymphatic endothelium in tumors is a result of the presence of VEGF-C, which acts on lymph vessels to enhance metastasis [18]. Other events include the transformation and cellular reprogramming of spindle-like lesional cells by Karposi sarcoma herpesvirus (KHSV)/human herpesvirus-8 (HHV8). Interestingly, gene profiling of KHSV infected lesional cells produce a signature similar to that derived from a lymphatic endothelial cell; targets that include Prox1, LYVE-1 and VEGFR-3. This suggests that Karposi sarcoma genetically mimics an expression pattern similar to a differentiated lymphatic endothelial cell [19-21].

## Conclusions

In conclusion, we extend the previous *in vitro *studies that characterized the molecular profile of reprogrammed vascular endothelial cells that overexpress the Prox1 transcription factor. Furthermore, we also demonstrate that the tight regulation of Prox1 is critical for normal embryonic lymphatic development. While the expression pattern of Prox1 in our model does not fully recapitulate the GFP expression found in the Tie1-GFP mouse model, it is clear that when Prox1 is overexpressed in our tissue specific model it results in aberrant lymph sac development, hemorrhaging, edema, anemia and ultimately, embryonic lethality. In a corresponding study, Johnson et al. demonstrated that Prox1 was essential for the maintenance of the lymphatic phenotype, where a global temporal deletion of Prox1 during mid-development effectively deprograms lymphatic structures to that of a vascular endothelial-like phenotype, assessed by surface markers such as Podoplanin and SLC [14]. Our data presented here complement the work by Johnston et al., whereby the tissue specific overexpression of Prox1 reprograms similar markers to a more lymphatic-like profile. Furthermore, we elucidate some of the molecular components that accompany vascular endothelial cell reprogramming involved in early lymphatic differentiation and development. The development of our unique model complements and extends the current viewpoint of Prox1 being an important initiator of lymphatic development via its ability to reprogram endothelial cells to a specific molecular profile.

## Methods

### Generation of mice

The Animal Care and Ethics Committee approved all animals and protocols that were used. The construction of the *tie1 *and *tie2 tTA *driver transgene has been previously described [22]. The *prox1 *cDNA was inserted into the *pTet*^*OS *^responder construct and transgenic animals were derived at the McGill Transgenic Facility, Montreal, Quebec, Canada. Driver and responder transgenic animals were bred to generate bigenic embryos. Embryos were genotyped and wild type, single and double transgenics in the presence of doxycycline was used as controls relative to double transgenics without doxycycline. Doxycycline treatment involved the addition of 100 μg/mL of doxycycline/5% sucrose in the drinking water and changed at least twice per week.

### Immunofluorescence and immunohistochemistry

Embryos were prepared by fixing in 4% paraformaldyhyde, followed by incubation in 30% sucrose and mounted in OCT for cryosectioning. Sections were treated with 0.5% TritonX-100/PBS and blocked in 5%BSA/10%goat serum prior to antibody incubation. Antibodies used were anti-Prox1 (102PA30, RDI), Podoplanin (clone 8.1.1) and VEGFR-3 (16-5988, eBiosciences). Immunohistochemistry was counterstained with Harris hematoxylin.

### RT-PCR analysis

Yolk sacs were placed in Trizol (GibcoBRL) and processed following manufacturers protocol. In brief, tissues were homogenized and 200 μL of chloroform was added per 1 mL Trizol. Following centrifugation at 10,000 × g for 15 minutes at 4°C, the upper phase was removed and 300 μL of 100% ethanol was added per 1 mL of Trizol. After 5 minutes incubation at room temperature, RNA was isolated by centrifugation at 2,000 × g for 5 minutes at 4°C. Proteins were then precipitated from the phenol-ethanol supernatant by 1.5 mL isopropyl alcohol per 1 mL Trizol. After 10 minutes incubation at room temperature, protein precipitate was isolated at 12,000 × g for 10 minutes at 4°C. Reverse transcriptase was performed as per manufacturers protocol (Qiagen). Negative control represents no template. PCR primers used were as follows:

mVEGFR-2 For: ATGAAATTGAGCTATCTGCC, Rev: CCACTGGATGTGGTGCAGGG

mNeuropilin-1For: GCAATAGCAAAAGAAGGTTT, Rev: ACCATGCCCAACAATCCAGA

mStat6 For: ATCCAGCTTCAGGCCCTGTC, Rev: TCTATCTGTGAGGAGCCATC

mCyclinE2 For: ATCCAGTCTACAGATTCCGA, Rev: ATCCAGTCTACAGATTCCGA

Prox1 For: ATGCCTGACCATGACAGC, Rev: GGGAAGCTTTTGCTTGCG

mJAM-1 For: CCGAGTGGAGTGGAAGTTCGTCC, Rev: AGGAACGACGAGGTCTGTTTGAATTC

mOccludin For: ACTTCAGGCAGCCTCGGTACAG, Rev: CTCCCGCAACTGGCATCTCTCTAA

mZO-1For: TCACAGGGCTCCTGGGTTTGGAT, Rev: CTAGTGACTGAATTTCTGAAATGTCATCT

GAPDH For: CTGCACCACCAACTGCTTAG, Rev: TCTCATCATACTTGGCAGGT

### Western analysis

Venous endothelial cells used in this study have been previously characterized [10]. Proteins were lysed in RIPA buffer for 30 minutes on ice (10 mM NaH_2_PO_4 _pH7.5, 150 mM NaCl, 1% NP-40, 0.1% SDS, 1% Sodium Deoxycholate, 10 mM NaF, 2 mM EDTA, Protease Inhibitor cocktail (Complete-EDTA free, Roche USA), and 10 mM sodium orthovanadate, cleared by centrifugation and the supernatants collected for further analysis. Equal amounts of lysates were resuspended with 2xSDS loading buffer and separated via SDS-PAGE. Proteins were transferred to PVDF, blocked with 3% milk/tris buffered saline, incubated with the appropriate primary and secondary antibody conjugated to horse radish peroxidase, and developed via enhanced chemiluminescence (Pierce). Antibodies used include Prox-1 (07-537, Upstate), CD31 (557355, BD Biosciences), ZO-1 (40-2300, Zymed) and β-actin (AC15, Sigma).

### Permeability assay

This protocol is a modification of the permeability assay by Chemicon (ECM640). To generate Prox1 overexpressing cells, bovine VECs [10] were transfected with a human Prox1 cDNA via Lipofectamine 2000 as per manufacturers protocol (Gibco). 2 × 10^5 ^cells were plated in a 3 μm pore size transwell with no substrate. Positive controls included 100 ng/mL of VEGF-A/25 mM EDTA in the top well. Also included in the top well was 250 μg/mL of FITC-Albumin to quantitate permeability through the cell monolayer. After the cells were incubated for 48 hours, passage of FITC-Albumin (250 μg/mL) into the bottom chamber was detected at an excitation/emission ratio of 488/530. n = 3, performed in triplicate.

### Statistical analysis

Data was presented as standard errors from the mean. Statistical significance was taken at *P *< 0.05 using an unpaired, two-tailed Student's *t-*test.

## Authors' contributions

HK was responsible for the experimental design, execution, and writing of this manuscript; VPKHN provided cell lines; TP constructed the transgenic construct; MC provided animal husbandry and technical support; KA Collaborator; DJD principle investigator. All authors read and approved the final manuscript.

## Supplementary Material

Additional file 1 **Prox1 overexpression in control and bigenic embryos**. (A and B) Control and (C and D) bigenic embryos at E10.5 were analyzed for Prox-1 expression (red) and smooth muscle actin (SMA, green). Of note, at this stage in development SMA is found to associate with the dorsal aorta (Panel C, arrow) but not the cardinal vein, thereby providing a simple landmark for identification. Significantly, (A) control embryos only display Prox-1 expression from the cardinal vein, however (C) DT embryos are positive for Prox-1 expression both the dorsal aorta (arrow) and the cardinal vein. (B and D) Sections have been counterstained with DAPI. CV: cardinal vein, DA: dorsal aorta, D: dorsal, L: lateral. Scale bar: A and B 100 μm, C and D 200 μm. Click here for file
