# Supplementary figures and images for: Embryonic vascular endothelial cells are malleable to reprogramming via Prox1 to a lymphatic gene signature
Source: BMC Dev Biol. 2010 Jun 28;10:72. doi: 10.1186/1471-213X-10-72 (PMC2909156; doi:10.1186/1471-213X-10-72)

Control

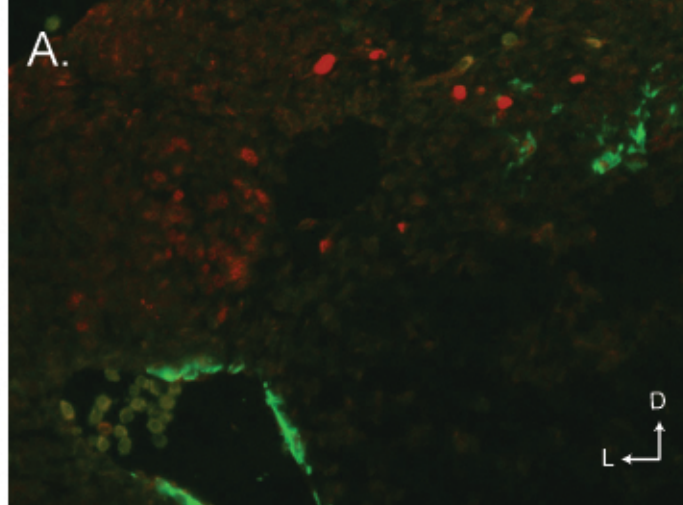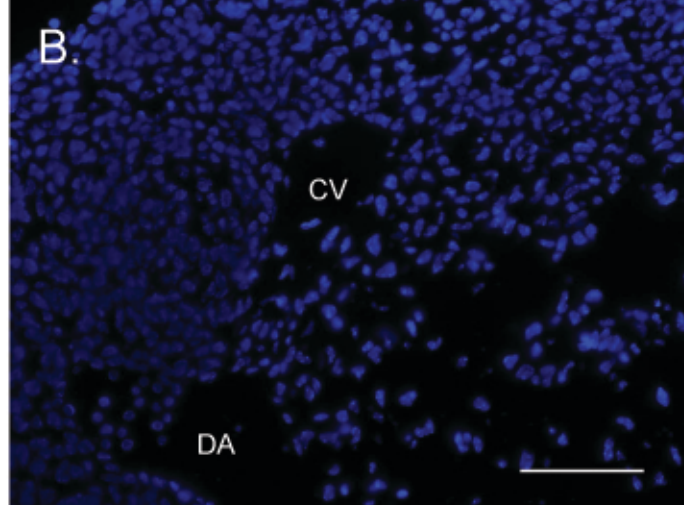

DT

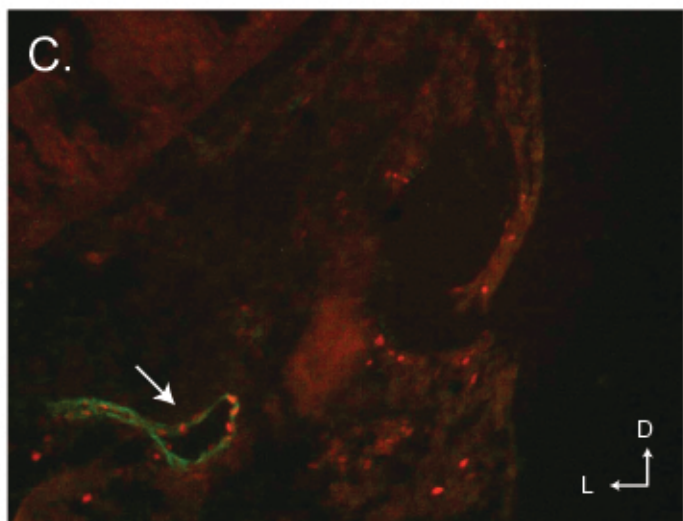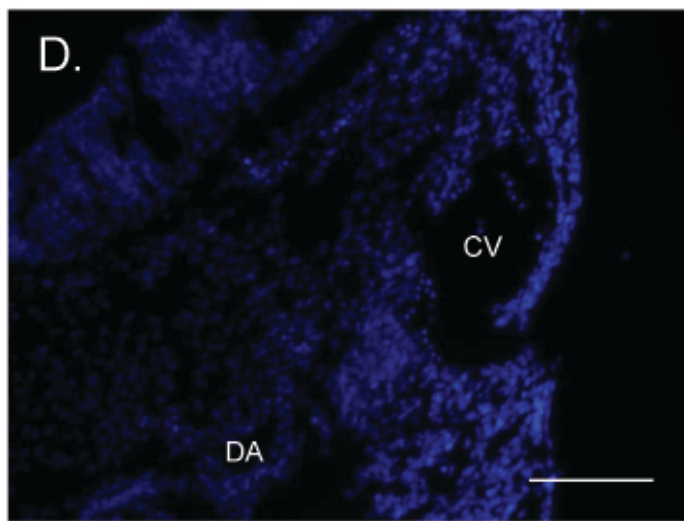

Prox-1

SMA

DAPI

Supplement: Additional file 1 — Prox1 overexpression in control and bigenic embryos. (A and B) Control and (C and D) bigenic embryos at E10.5 were analyzed for Prox-1 expression (red) and smooth muscle actin (SMA, green). Of note, at this stage in development SMA is found to associate with the dorsal aorta (Panel C, arrow) but not the cardinal vein, thereby providing a simple landmark for identification. Significantly, (A) control embryos only display Prox-1 expression from the cardinal vein, however (C) DT embryos are positive for Prox-1 expression both the dorsal aorta (arrow) and the cardinal vein. (B and D) Sections have been counterstained with DAPI. CV: cardinal vein, DA: dorsal aorta, D: dorsal, L: lateral. Scale bar: A and B 100 μm, C and D 200 μm. [file 1471-213X-10-72-S1.PDF]
